# Low-dose CBCT reconstruction via joint non-local total variation denoising and cubic B-spline interpolation

**DOI:** 10.1038/s41598-021-83266-1

**Published:** 2021-02-11

**Authors:** Ho Lee, Jiyoung Park, Yeonho Choi, Kyung Ran Park, Byung Jun Min, Ik Jae Lee

**Affiliations:** 1 Department of Radiation Oncology, Gangnam Severance Hospital, Yonsei University College of Medicine, Seoul, Korea; 2 Department of Radiation Oncology, Kosin University College of Medicine, Busan, Korea; 3 Department of Radiation Oncology, Chungbuk National University Hospital, Cheongju, Korea

**Keywords:** Biomedical engineering, Imaging techniques, Dental equipment

## Abstract

This study develops an improved Feldkamp–Davis–Kress (FDK) reconstruction algorithm using non-local total variation (NLTV) denoising and a cubic B-spline interpolation-based backprojector to enhance the image quality of low-dose cone-beam computed tomography (CBCT). The NLTV objective function is minimized on all log-transformed projections using steepest gradient descent optimization with an adaptive control of the step size to augment the difference between a real structure and noise. The proposed algorithm was evaluated using a phantom data set acquired from a low-dose protocol with lower milliampere-seconds (mAs).The combination of NLTV minimization and cubic B-spline interpolation rendered the enhanced reconstruction images with significantly reduced noise compared to conventional FDK and local total variation with anisotropic penalty. The artifacts were remarkably suppressed in the reconstructed images. Quantitative analysis of reconstruction images using low-dose projections acquired from low mAs showed a contrast-to-noise ratio with spatial resolution comparable to images reconstructed using projections acquired from high mAs. The proposed approach produced the lowest RMSE and the highest correlation. These results indicate that the proposed algorithm enables application of the conventional FDK algorithm for low mAs image reconstruction in low-dose CBCT imaging, thereby eliminating the need for more computationally demanding algorithms. The substantial reductions in radiation exposure associated with the low mAs projection acquisition may facilitate wider practical applications of daily online CBCT imaging.

## Introduction

Volumetric imaging, such as cone-beam computed tomography (CBCT) or megavoltage CT, is being rapidly adopted as a common modality in image guided radiation therapy (IGRT)^1–4^. In particular, CBCT mounted on the gantry of a linear accelerator is scanned to improve the target accuracy and precision by minimizing anatomic deviations prior to the beginning of each fraction^5^. The reduction of these setup uncertainties permits the safe implementation of a noticeable dose falloff between the boundaries of the planned treatment volume and the surrounding organs at risk. Moreover, several studies have utilized CBCT images on every treatment day for dose calculation to verify if the actual delivered dose deviates from the planned dose distributions^6,7^. In current protocols, the dose amount from each CBCT scan is approximately 3 cGy for parts of the skin and as high as 5 cGy for inside the body. When a patient is imaged daily during the course of radiotherapy that lasts four to six weeks^8^, the accumulated dose exceeds 100 cGy at the region inside the field of view (FOV). Although this amount may seem adequate in the context of radiotherapy, it cannot be ignored. If a judicious image quality can be obtained with a low dose, it is clear to comply the directions. Consequently, it is vital to develop an efficient reconstruction algorithm for daily CBCT imaging with dose reduction.

The most straightforward way to reduce dose of a CBCT system is to use either a lower mAs of the projection data or a shorter exposure time per projection^9^. Utilization of these approaches leads to excessive noise in the reconstructed images. The iterative and analytical reconstruction algorithms can be considered via these strategies. Compressed sensing (CS) theory in iterative reconstruction methods has been commonly utilized to generate a reconstructed image with satisfactory quality from a highly undersampled number of projections below the Nyquist rate. The CS method involves minimizing a total variation objective function subject to data fidelity and physical constraints through constrained or unconstrained optimization methods. Its efficacy has been improved by incorporating prior images into an objective function^10,11^. However, the use of such approaches is primarily limited by their high computational demands in practical applications, because the reprojection and backprojection steps are iteratively performed to calculate the fidelity between measured and estimated projections. Meanwhile, analytical reconstruction algorithms such as Feldkamp–Davis–Kress (FDK)^12^ enable high speed performance by using only one filtering process and one backprojection process. These approaches are required when the projection data are complete (with the number of projections fully available) because its performance is greatly influenced by the filtering process. However, the excess noise caused by the low mAs protocol leads to significantly degraded CBCT image qualities when they are attempted using FDK with conventional filtering. An efficient denoising technique^13,14^ is thus required to enhance the difference in the signals between real structures and unwanted noise for better CBCT image quality. Recently, several deep learning-based reconstruction approaches have been reported. Deep learning is a new concept with respect to image quality optimization without hand-engineering mapping functions. One way to use deep learning methods for image-to-image conversion is supervised training based on paired images^15–18^. These approaches employ deep neural networks in the image or projection domain^19^. Another way is unsupervised training using unpaired images, such as cycle-generative adversarial networks^20^. For supervised learning, it is difficult to obtain anatomically exact matching paired images. Even for unsupervised learning, sufficient training data are required to achieve good results. However, the lack of injectivity and stability of deep learning approaches for image reconstruction poses potential issues^21^. Therefore this type of deep learning algorithm should be further explored.

In this study, an improved FDK-based CBCT reconstruction algorithm is proposed based on non-local total variation (NLTV) filtration and cubic B-spline interpolation for low mAs of projection data. The proposed approach employs the framework of the standard FDK algorithm. The NLTV denoising process is applied on the log-transformed projection data. Minimizing the NLTV objective function using the steepest gradient descent optimization with an adaptive control of the step size indicates that edges having high contrast relative to the surroundings are preserved and noisy pixels having a low contrast are smoothed. With filtered projections that apply a ramp filtering on NLTV minimized projections, a voxel-driven backprojector based on a cubic B-spline interpolation is utilized to generate the reconstructed images. The effectiveness of the proposed method is demonstrated using phantom studies.

## Methods

**Figure 1:**
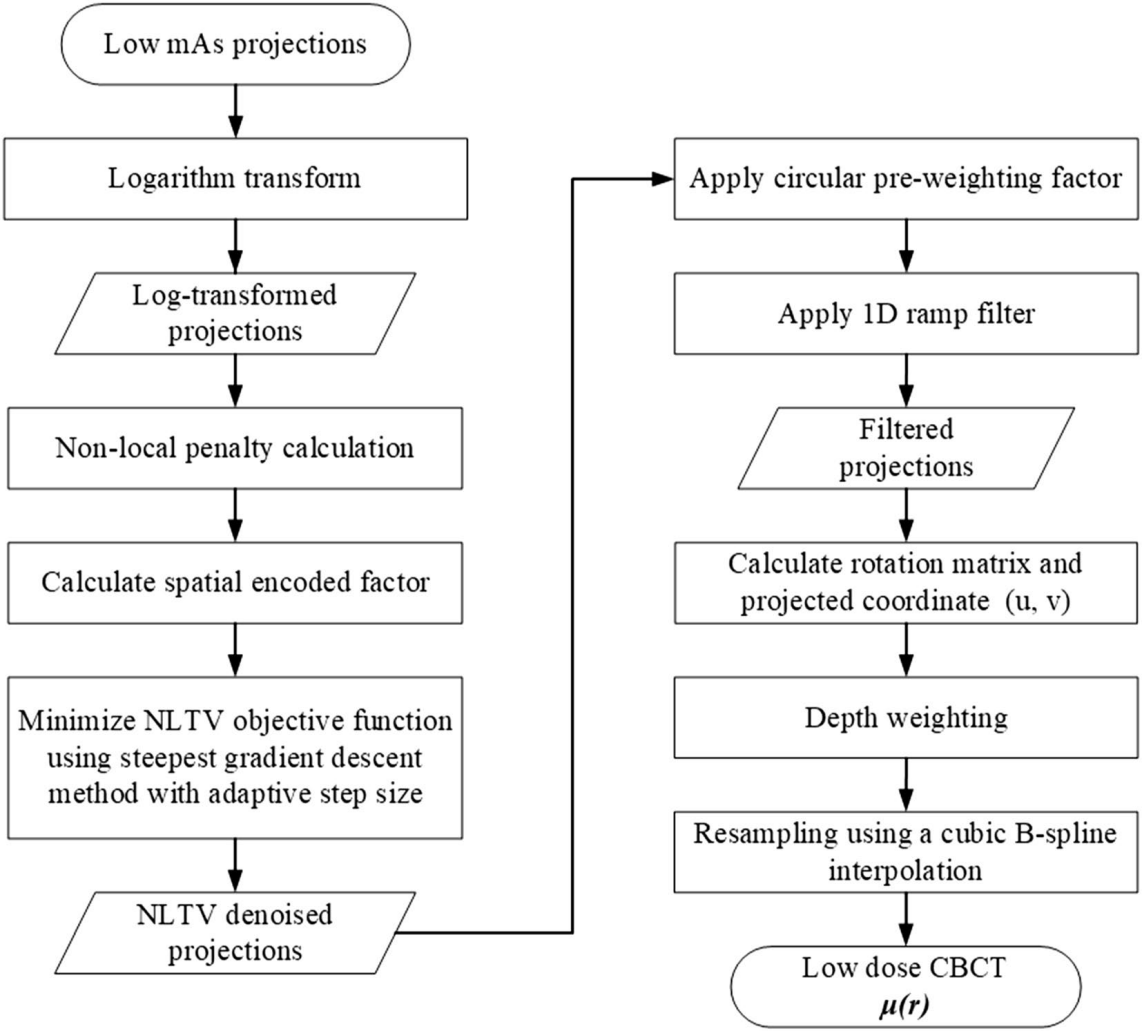
Overview of the proposed method for low-dose CBCT imaging.

Figure 1 shows a flowchart of the proposed algorithm using joint NLTV denoising-based filtration and a cubic B-spline interpolation-based backprojector to enhance the image quality of low-dose CBCT from projection data acquired from a low-dose protocol with lower mAs. The NLTV denoising is used to increase the difference between the actual structure and the noise of the projection data. The cubic B-spline interpolation is applied to update the voxels in the reconstructed image by resampling 16 adjacent pixels in the projection domain during backprojection.

### Filtration using non-local total variation

A total variation^22^ denoising process on log-transformed projections is applied to enhance the difference in the signals between striking features and unwanted noise by similarities between non-local patches^23–25^. Minimizing the NLTV objective function indicates that edges having high contrast relative to the surroundings are preserved and noisy voxels having a low contrast are smoothed. The non-local penalty with different weights for neighbors of the same distance in the TV object function is written as follows:

1 $$R\left( P \right) = \mathop \sum \limits_{j} R\left( {P_{j} } \right) = \mathop \sum \limits_{j} w_{j} D\left( {P_{j} } \right),$$

2 $$w_{j} = \mathop \sum \limits_{{i \in {\Omega }}} exp\left( { - \left( {P_{j} /\tau } \right)^{\varepsilon } \frac{{\mathop \sum \nolimits_{k = - a}^{k = a} G\left( k \right)\left( {P_{{\left( {j + k} \right)}} - P_{{\left( {i + k} \right)}} } \right)^{2} }}{{2h_{0}^{2} }}} \right),$$

3 $$D\left( {P_{j} } \right) = \sqrt {\left( {P_{{\left( {u,v} \right)}} - P_{{\left( {u - 1,v} \right)}} } \right)^{2} + \left( {P_{{\left( {u,v} \right)}} - P_{{\left( {u,v - 1} \right)}} } \right)^{2} } ,$$

where index *j* identifies the index of the pixel element in the projection and $P_{{\left( {u,v} \right)}}$ is the pixel element at the 2D position (u, v). The search set for the non-local areas is defined as *i* ∈ Ω and *w*_*j*_ represents the weights between the current voxel *j* and the voxels in Ω. G(k) is the Gaussian kernel with a patch size of ((2a + 1) × (2a + 1)), and *k* identifies the index of the patch. In this work, the patch of the Gaussian kernel is defined to be 5 × 5 with unit variance; the non-local search area is 21 × 21 with unit variance, and the filtering parameter *h*_*0*_ is set to 90% of the cumulative distribution function (CDF) histogram created by accumulating the gradient magnitude at each pixel of the log-transformed projection data. $\left( {P_{j} /\tau } \right)^{\varepsilon }$ is the spatially encoded factor for the *j-*th pixel, which has two hyperparameters τ and $\varepsilon$^24^. Its role is to reduce the weighted averaging effect in high intensity regions to maintain the contrast. The parameter τ is the normalization factor to make the $P_{j} /\tau$ ratio exceed 1. In this study, the parameter τ was set to 90% of the CDF histogram created by accumulating the intensity at each pixel of the log-transformed projection data. The parameter $\varepsilon$ (in this study, we used $\varepsilon$ = 3) is the scaling factor to make smaller weights for higher intensities, thereby reducing the blurring effect in high intensity regions.

The NLTV objective function of Eq. (2) is minimized by the steepest gradient descent method with an adaptive step size, which is expressed as follows:

4 $$P_{j}^{t + 1} = P_{j}^{t} - \lambda \frac{{\nabla R\left( {P_{j} } \right)}}{{\left| {\nabla R\left( P \right)} \right|}},$$

5 $$\lambda = \gamma \sqrt {\mathop \sum \limits_{j} P_{j}^{2} } ,$$

where λ is an adaptive parameter that controls the step size so that the smoothing degree is decreased according to an advancement in the iteration steps. By using the square root of all pixel elements updated in each steepest gradient descent step, this parameter is forced to progressively smaller values with an increase in the number of iterations. To avoid a local minimization due to an abrupt change, we use a scaling parameter $$\gamma$$ and set it to an initial value of 1.0. If $$R\left( P \right)$$ calculated at the current iteration step is larger than that at the previous one, this value is linearly decreased by multiplying a constant value ($$r_{red} = 0.8)$$. $$\nabla {\text{R}}\left( {P_{j} } \right)$$ is the gradient of the objective function $$R\left( P \right)$$ at the *j-*th indexed pixel. The root-sum-square of the gradient calculated at all pixels, $$\left| {\nabla R\left( P \right)} \right|$$, is required for the normalized gradient calculation. For clarity, we provide the following expression:

6 $$\nabla R\left( {P_{j} } \right) = \frac{\partial R\left( P \right)}{{\partial P_{j} }} = \frac{\partial R\left( P \right)}{{\partial P_{{\left( {u,v} \right)}} }} = \left( \begin{gathered} w_{{\left( {u,v} \right)}} \frac{{2P_{{\left( {u,v} \right)}} - P_{{\left( {u - 1,v} \right)}} - P_{{\left( {u,v - 1} \right)}} }}{{\sqrt {\left( {P_{{\left( {u,v} \right)}} - P_{{\left( {u - 1,v} \right)}} } \right)^{2} + \left( {P_{{\left( {u,v} \right)}} - P_{{\left( {u,v - 1} \right)}} } \right)^{2} } }} \hfill \\ + w_{{\left( {u + 1,v} \right)}} \frac{{P_{{\left( {u,v} \right)}} - P_{{\left( {u + 1,v} \right)}} }}{{\sqrt {\left( {P_{{\left( {u + 1,v} \right)}} - P_{{\left( {u,v} \right)}} } \right)^{2} + \left( {P_{{\left( {u + 1,v} \right)}} - P_{{\left( {u + 1,v - 1} \right)}} } \right)^{2} } }} \hfill \\ + w_{{\left( {u,v + 1} \right)}} \frac{{P_{{\left( {u,v} \right)}} - P_{{\left( {u,v + 1} \right)}} }}{{\sqrt {\left( {P_{{\left( {u,v + 1} \right)}} - P_{{\left( {u - 1,v + 1} \right)}} } \right)^{2} + \left( {P_{{\left( {u,v + 1} \right)}} - P_{{\left( {u,v} \right)}} } \right)^{2} } }} \hfill \\ \end{gathered} \right),$$

7 $$\left| {\nabla R\left( P \right)} \right| = \sqrt {\mathop \sum \limits_{j} \left( {\nabla R\left( {P_{j} } \right)} \right)^{2} } ,$$

The optimal number of iterations for the steepest gradient descent step was fine-tuned so that the noisy pixels were minimized while preserving the striking features. In this study, the number of iterations for the steepest gradient descent step was set to ten. In considering all the components mentioned above, we present the pseudo-code of NLTV-based filtration as in Algorithm 1.

**Figure 2:**
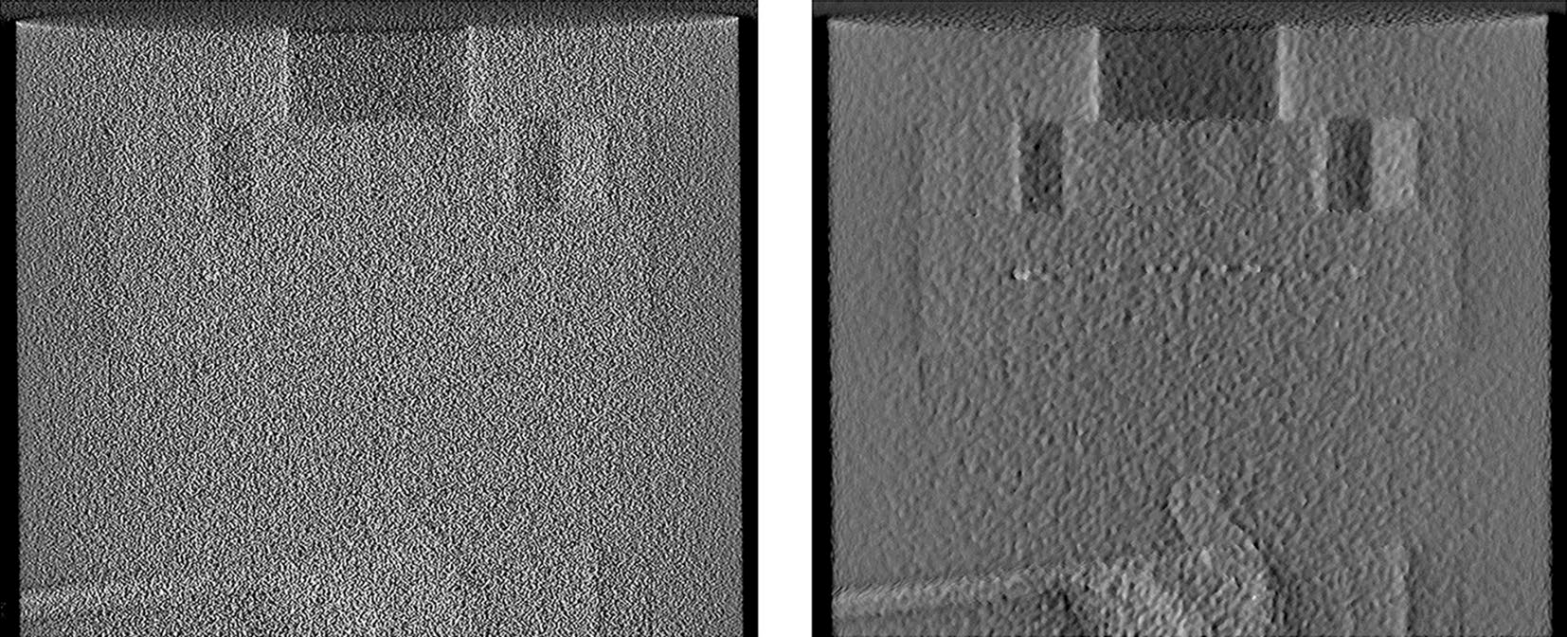
Comparison of filtered projection without (left) and with (right) the NLTV denoising algorithm.


A circular pre-weighting factor was then applied on each NLTV denoised projection data to avoid the intensity drop due to the cone angle effect. A 1D ramp filter was then applied to suppress the highest spatial frequency. For the ramp filter, we used the product of the SL filter and raised the cosine window function in the frequency domain. Each line parallel to the u-direction of the preweighted projection data was converted into the frequency domain via 1D Fourier transformation. The Fourier transformed values were multiplied by the ramp filter. By applying an inverse Fourier transformation, we could obtain the filtered projection data with the reduced impulse noise. Figure 2 depicts the differences between filtered projections before and after applying the NLTV denoising algorithm.

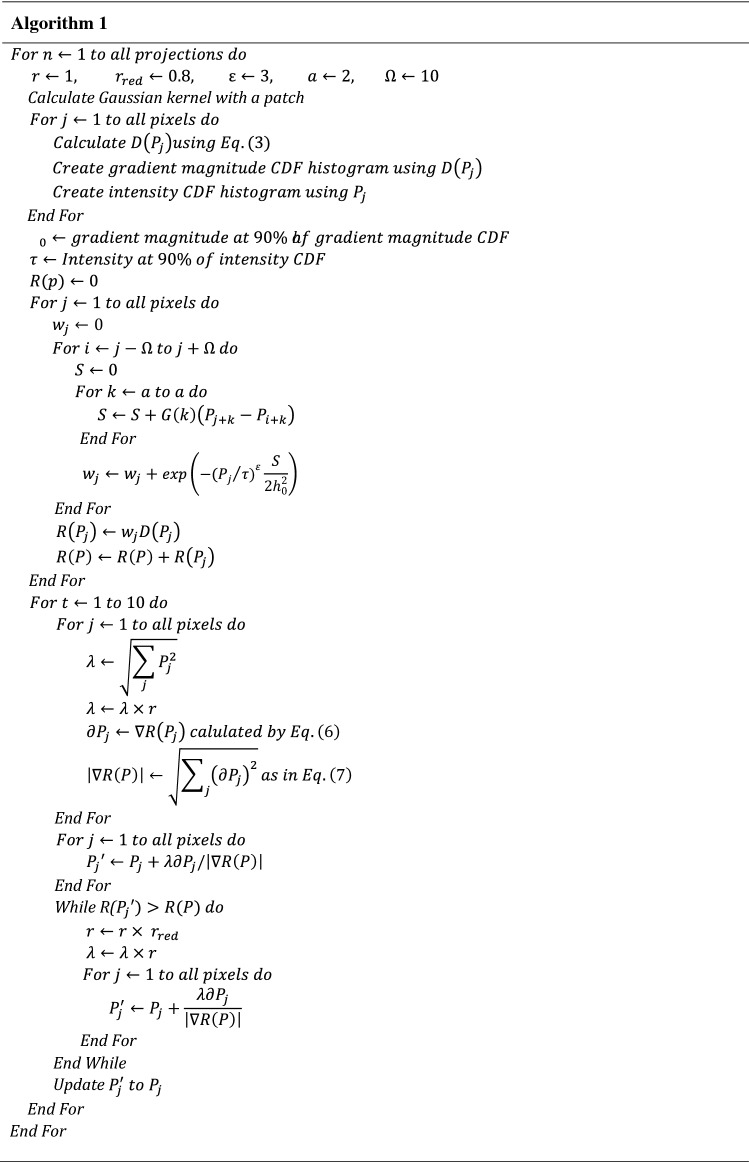


### Voxel-driven backprojector based on cubic B-spline interpolation

**Figure 3:**
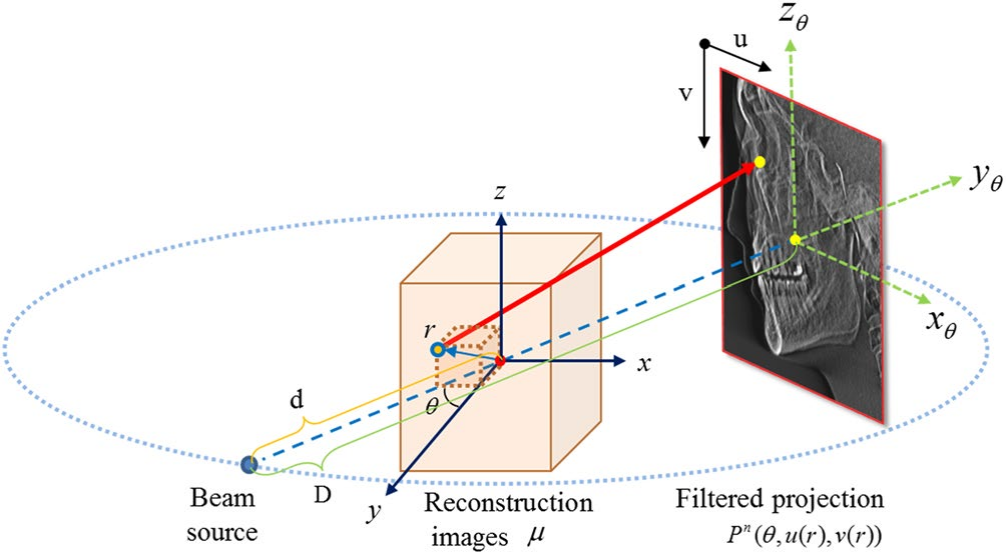
Geometry of the projection data and notations for the voxel-driven backprojection step.

Figure 3 shows the geometric information of the projection data and notations for voxel-driven backprojection calculation using the filtered projection data.

Given a position vector ***r ***(or *r*_*x*_, *r*_*y*_, *r*_*z*_) of any voxel, corresponding positions (u(r) and v(r)) in the n-th projection data at a given angle θ are obtained as follows:

8 $$P\left( {\theta , r} \right) = P^{n} \left( {\theta ,u\left( r \right),v\left( r \right)} \right),$$

where

9 $$u\left( r \right) = \frac{{r_{x\theta } }}{{d + r_{y\theta } }},$$

10 $$v\left( r \right) = \frac{{r_{z\theta } }}{{d + r_{y\theta } }},$$

Here, *d* is the source-to-axis distance, and the rotation vectors $\left[ {r_{x\theta } ,r_{y\theta } ,r_{z\theta } } \right]^{T}$ are calculated by applying the rotation matrix through a given angle θ from the origin of the reconstruction images in the following manner:

11 $$\left( {\begin{array}{*{20}c} {r_{x\theta } } \\ {r_{y\theta } } \\ {r_{z\theta } } \\ \end{array} } \right) = \left( {\begin{array}{*{20}c} {cos\theta } & { - sin\theta } & 0 \\ {sin\theta } & {cos\theta } & 0 \\ 0 & 0 & 1 \\ \end{array} } \right)\left( {\begin{array}{*{20}c} {r_{x} } \\ {r_{y} } \\ {r_{z} } \\ \end{array} } \right),$$

**Figure 4:**
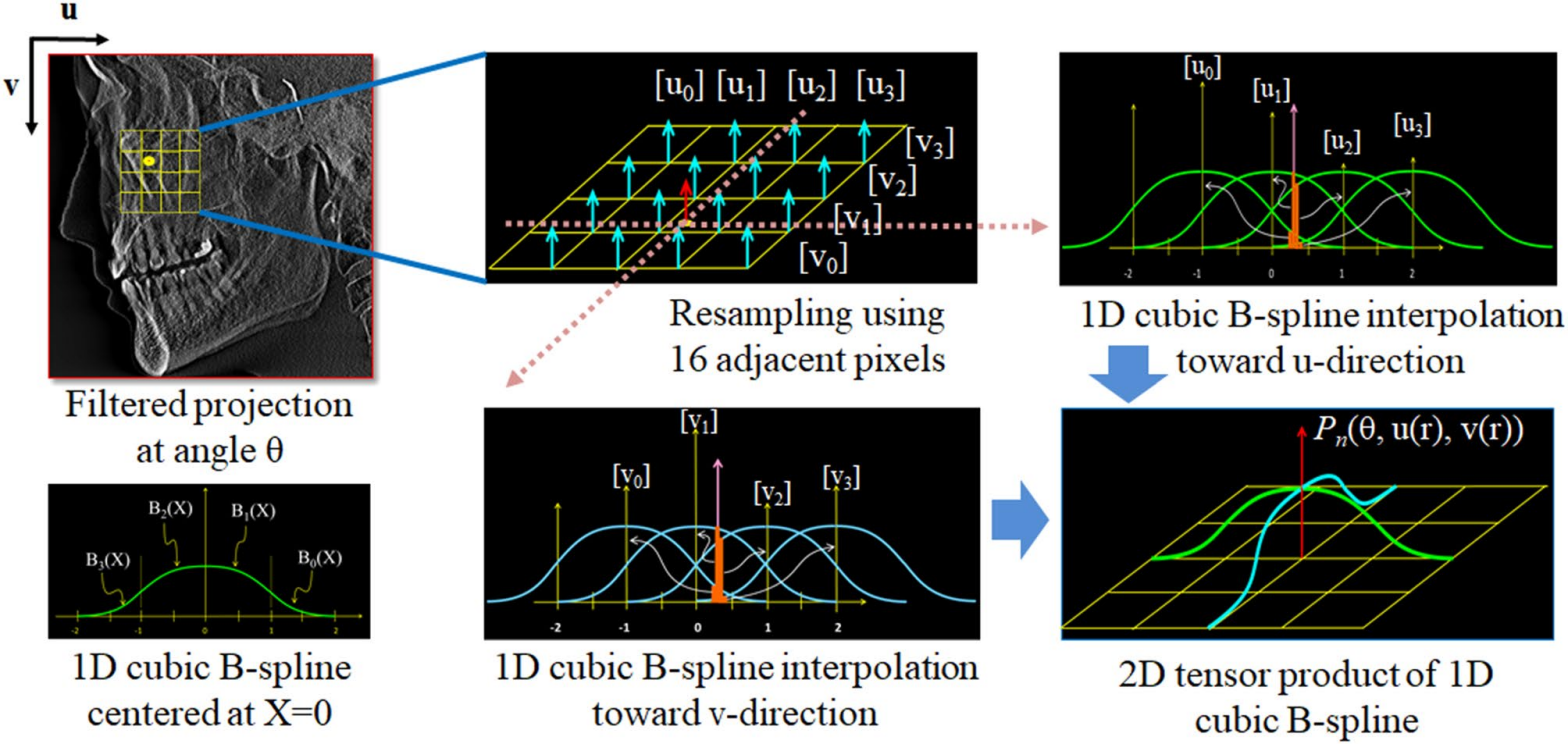
Resampling process using 2D tensor product of 1D cubic B-spline interpolation from n-th filtered projection.

The final attenuation coefficient value, *μ*(*r)*, at a position vector *r* is weighted and accumulated as

12 $$\mu \left( r \right) = \mu \left( {r_{x} ,r_{y} ,r_{z} } \right) = \frac{\pi }{N}\mathop \sum \limits_{n = 1}^{N} \left( {w\left( r \right)P^{n} \left( {\theta ,u\left( r \right),v\left( r \right)} \right)} \right),$$

13 $$w\left( r \right) = d^{2} /\left( {d + r_{y\theta } } \right)^{2} ,$$

where *w*(*r*) is the depth weighting and *N* is the total number of projection data. Every voxel has measurements in all filtered projection data and is normalized by a uniform factor *N* in the backprojection step. As shown in Fig. 4, $P^{n} \left( {\theta ,u\left( r \right),v\left( r \right)} \right)$ is represented as the 2D tensor product of a 1D cubic B-spline:

14 $$P^{n} \left( {\theta ,u\left( r \right),v\left( r \right)} \right) = \mathop \sum \limits_{p = 0}^{3} \mathop \sum \limits_{q = 0}^{3} B_{p} \left( {u - \left[ {u_{p} } \right]} \right)B_{q} \left( {v - \left[ {v_{q} } \right]} \right)P^{n} \left( {\theta ,\left[ {u_{p} } \right],\left[ {v_{q} } \right]} \right),$$

where $u_{p} = u + p - 1$, $v_{q} = v + q - 1$, and $\left[ { \cdot } \right]$ is the floor function that maps a real number to the largest previous integer. $B_{p}$ and $B_{q}$ correspond to the following cubic B-spline basis functions centered at X = 0:

15 $$\begin{aligned} B_{0} \left( X \right) & = \left( {2 - {\text{X}}} \right)^{3} /6\quad \quad \quad \quad \quad if\quad 1 \le X \le 2 \\ B_{1} \left( X \right) & = \left( {3{\text{X}}^{3} - 6{\text{X}}^{2} + 4} \right)/6\quad \quad if\quad 0 \le X < 1 \\ B_{2} \left( X \right) & = \left( { - 3{\text{X}}^{3} - 6{\text{X}}^{2} + 4} \right)/6\quad \,\,if\quad - 1 \le X < 0 \\ B_{3} \left( X \right) & = \left( {2 + {\text{X}}} \right)^{3} /6\quad \quad \quad \quad \quad if\quad - 2 \le X < - 1. \\ \end{aligned}$$

In considering all the components mentioned above, we present the pseudo-code of cubic B-spline interpolation-based backprojector as in Algorithm 2.
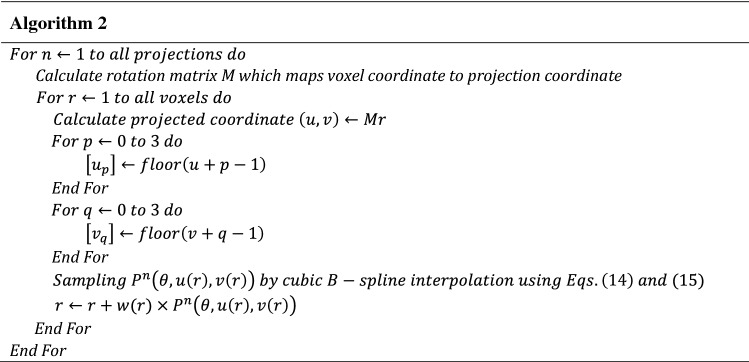


### Experimental studies

The Catphan503 (Phantom Laboratory, Salem, NY) was scanned using an Infinity™ linear accelerator system with XVI R5.0 (Elekta Limited, Stockholm, Sweden). The phantom includes various modules for assessing the quality of 3D images. As the gantry rotates around the phantom, a series of CBCT projections were acquired with 360° rotation under a small FOV protocol using a S20 collimator and an F0 filter. The number of full scan projections was approximately 665. The size of each projection acquired on the image receptor was 409.6 × 409.6 mm^2^, containing 1024 × 1024 pixels. The distance from the source to the detector and the distance from the source to the axis were 1536 mm and 1000 mm, respectively. The low-dose acquisition protocol had 100kVp with 0.1 mAs during each projection. Projections with different mAs settings were also acquired for comparison. The reconstructed image of the phantom was generated with two resolution settings: high resolution CBCT (512 × 512 × 200 voxels as well as 0.5 × 0.5 × 0.5 mm^3^ per each voxel) and low resolution CBCT (256 × 256 × 100 voxels as well as 1.0 × 1.0 × 1.0 mm^3^ per each voxel). For comparison, a high-dose CBCT was created as a benchmark image by applying a conventional FDK algorithm with projections acquired using increased mAs setting (100 kVp and 1.6 mAs). The Hounsfield unit (HU) conversion was performed for all reconstructed images. The CBCT image quality was quantified using three metrics (CNR, HU accuracy, and correlation).

The accuracy of the HU number was assessed using the entire area of the CTP404 module of the phantom. It has seven sensitometry inserts and the RMSE is calculated. The correlation and the CNR were also measured using the same module. The correlation was calculated to evaluate the overall differences of the reconstructed CBCT image versus benchmark CBCT images. For the RMSE and correlation measurement, a circular area was selected to be measured only inside the phantom excluding the air area outside the phantom. For the CNR measurement, the mean HU value and standard deviation were recorded by selecting seven ROIs within the density insert and a central ROI.

## Results

**Figure 5:**
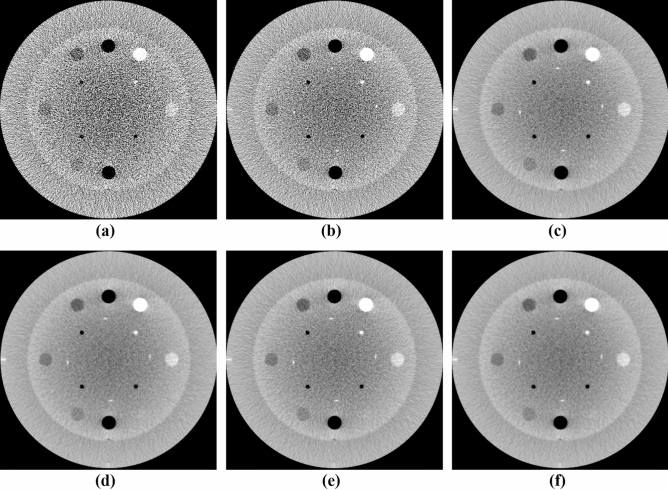
Comparison between the same views of the high resolution reconstruction image generated by applying FDK with (**a**) SL, (**b**) ATV, (**c**) NLTV, (**d**) SL + cubic B-spline, (**e**) ATV + cubic B-spline, and (**f**) NLTV + cubic B-spline using the Catphan503 phantom. These images are displayed at window (width and level) settings of (975, 0) HU.

**Figure 6:**
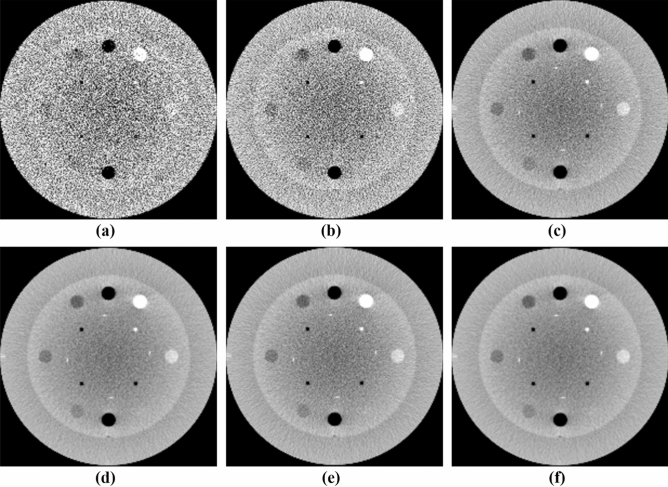
Comparison between the same views of the low resolution reconstruction image generated by applying FDK with (**a**) SL, (**b**) ATV, (**c**) NLTV, (**d**) SL + cubic B-spline, (**e**) ATV + cubic B-spline, and (**f**) NLTV + cubic B-spline using the Catphan503 phantom. These images are displayed at window (width and level) settings of (975, 0) HU.

The performance of the NLTV denoised FDK reconstruction algorithm was evaluated with the Catphan503 phantom and compared with the Shepp–Logan (SL)-filtered FDK and anisotropic total variation (ATV)- denoised FDK^26^. The ATV combines the edge stopping function used locally in an anisotropic diffusion filter^27^ for both image enhancement and noise reduction. Figures 5 and 6 show a representative slice of the high resolution CBCT (512 × 512 × 200 voxels) and low resolution CBCT (256 × 256 × 100 voxels) reconstructed from FDKs with SL, ATV, and NLTV, as well as when adding the voxel-driven backprojector based on cubic B-spline interpolation on filtered projections with SL, ATV, and NLTV, respectively. The artifacts were significantly suppressed in images reconstructed by the proposed NLTV denoising compared to the other two algorithms. The CBCT images resampled with the cubic B-spline interpolation using 16 pixels (4 × 4) were smoother and had fewer interpolation artifacts, resulting in slightly improved reconstruction accuracy for SL, ATV, and NLTV. The effect of this image quality improvement was the same for low resolution CBCT. In particular, the cubic B-spline interpolation still visibly achieved substantial gains on all SL, ATV, and NLTV.

In addition, we calculated the contrast-to-noise ratio (CNR) at a selected region of interest (ROI) in the reconstructed image. It was possible to conduct a comparison of the relative image contrast between the corresponding regions. Tables 1 and 2 provide a comparison of CNRs at seven ROIs in the high resolution and low resolution reconstructed CBCT images obtained by six FDK algorithms. The proposed NLTV denoising shows a better CNR in all ROIs compared to other two algorithms. Adding the cubic B-spline interpolation process slightly improved the CNR to higher values for SL, ATV, and NLTV. Tables 3 and 4 list two quantitative measures (root-mean-square error (RMSE) and correlation) obtained from the high resolution and low resolution reconstructed images based on the six FDK methods. The errors were computed against full projections with a high mAs acquisition protocol (40 mA and 40 ms). The NLTV elicited a lower RMSE and a higher correlation compared to the other algorithms. Adding the cubic B-spline interpolation produced the best results for the Catphan503 phantom with a low noise scenario. Even in the SL and ATV, the cubic B-spline interpolation could yield a lower RMSE and a higher correlation. Our proposed NLTV with the cubic B-spline interpolation produced the lowest RMSE and the highest correlation. This relative superiority of the proposed method was the same for the low resolution CBCT. The low resolution CBCT doubled the voxel size compared to the high resolution CBCT, leading to inferior feature details. This resulted in larger RMSE values and smaller CNR values in the quantitative analysis.

**Table 1:** Comparison of CNRs at seven ROIs in the high resolution reconstructed image generated by six FDK algorithms with low-dose projection data of the Catphan503 phantom.

ROI	Material of insert	SL	ATV	NLTV	SL + cubic B-spline	ATV + cubic B-spline	NLTV + cubic B-spline
1	Delrin™	2.0	8.7	14.4	3.5	11.6	16.4
2	Teflon	4.2	14.3	23.6	7.0	18.2	25.9
3	Air	4.2	20.5	34.8	8.0	28.1	39.8
4	PMP	0.4	1.8	3.2	0.7	2.5	3.8
5	LDPE	0.1	0.2	0.4	0.1	0.3	0.5
6	Polystyrene	0.1	0.8	1.4	0.3	1.1	1.6
7	Air	4.3	21.0	35.9	8.3	29.1	41.9

**Table 2:** Comparison of CNRs at seven ROIs in the low resolution reconstructed image generated by six FDK algorithms with low-dose projection data of the Catphan503 phantom.

ROI	Material of insert	SL	ATV	NLTV	SL + cubic B-spline	ATV + cubic B-spline	NLTV + cubic B-spline
1	Delrin™	1.7	7.6	11.3	3.4	10.1	13.7
2	Teflon	4.2	13.2	20.6	6.5	17.8	24.2
3	Air	4.7	19.3	29.9	8.3	25.5	33.7
4	PMP	0.5	2.1	3.4	1.0	2.9	4.1
5	LDPE	0.1	0.3	0.4	0.1	0.3	0.5
6	Polystyrene	0.1	0.4	0.9	0.2	0.6	1.0
7	Air	4.0	19.7	31.6	7.8	27.3	37.1

**Table 3:** Quantitative comparisons using two metrics in the high resolution reconstruction image generated by six FDK algorithms with low-dose projection data of the Catphan503 phantom.

	SL	ATV	NLTV	SL + cubic B-spline	ATV + cubic B-spline	NLTV + cubic B-spline
RMSE	181.0	69.0	62.4	112.6	64.3	61.4
Correlation	0.38	0.76	0.82	0.57	0.80	0.83

**Table 4:** Quantitative comparisons using two metrics in the low resolution reconstruction image generated by six FDK algorithms with low-dose projection data of the Catphan503 phantom.

	SL	ATV	NLTV	SL + cubic B-spline	ATV + cubic B-spline	NLTV + cubic B-spline
RMSE	207.0	136.8	133.1	155.9	135.3	132.2
Correlation	0.43	0.77	0.82	0.57	0.80	0.82

Table 5 compares the computation times between the proposed method and other methods used in Figs. 5 and 6. Compared to SL and ATV, the computation time using the proposed NLTV denoising was approximately 14.5 times and 5.1 times longer for the high resolution CBCT generation and 17.3 times and 5.4 times longer for the low resolution CBCT. It was influenced by the size and number of projections because NLTV was applied to the projection data. Thus, the difference in calculation time due to high and low resolutions was not large. On the other hand, the calculation time before and after applying the cubic B-spline interpolation required approximately 90 s longer for high resolution CBCT and 10 s longer for the low resolution CBCT. Since the cubic B-spline interpolation was applied after estimating the projection pixel for each voxel in the reconstructed volume, it was affected by the reconstruction volume size. This resulted in a large difference in the calculation time between the high and low resolution CBCTs.

**Table 5:** Computation time (seconds) of six FDK algorithms when creating high resolution and low resolution CBCTs of the Catphan503 phantom.

	SL	ATV	NLTV	SL + cubic B-spline	ATV + cubic B-spline	NLTV + cubic B-spline
High resolution CBCT	95.8	269.4	1398.6	192.0	355.2	1485.8
Low resolution CBCT	79.7	251.4	1379.4	91.1	261.9	1390.0

**Figure 7:**
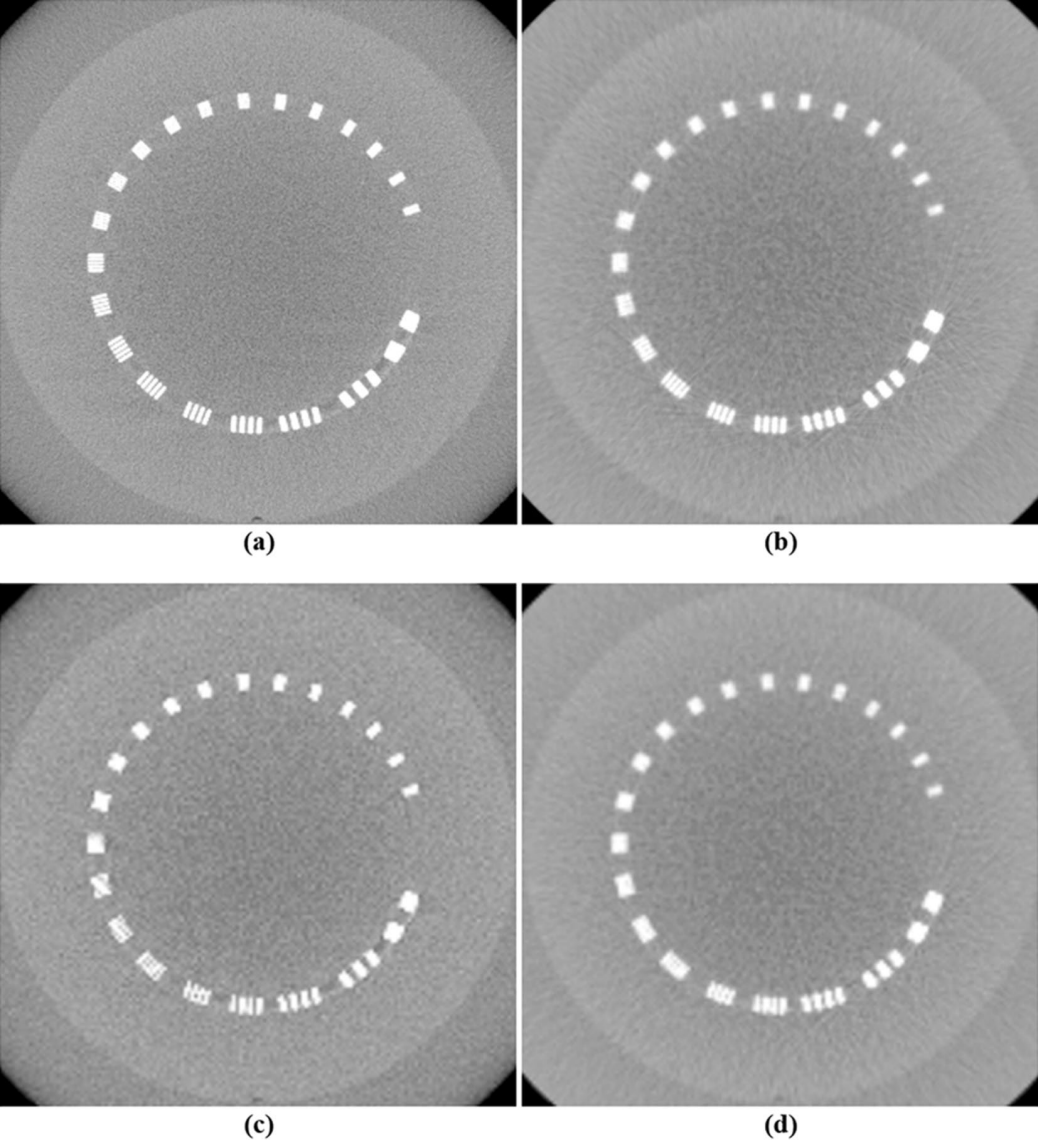
Comparison of a representative slice of the reconstruction image generated by applying FDK using high mAs projections and FDK with NLTV + cubic B-spline interpolation using low mAs projections. (**a**) high resolution benchmark image, (**b**) high resolution CBCT produced by the proposed method, (**c**) low resolution benchmark image, and (**d**) low resolution CBCT produced by the proposed method. These images are displayed at window (width and level) settings of (2000, 0) HU.

Figure 7 shows the proposed method with projections acquired at low mAs levels and the conventional FDK algorithm with projections acquired at high mAs levels (40 mA and 40 ms). These images enable us to qualitatively judge the correspondence of line bars between the two reconstruction images. It was observed that the proposed method shows a spatial resolution comparable to that of high-dose CBCT imaging through visual inspection. Even with a high voxel size and low resolution, the visual effect was better than a low resolution benchmark image with false artificial traces.

**Figure 8:**
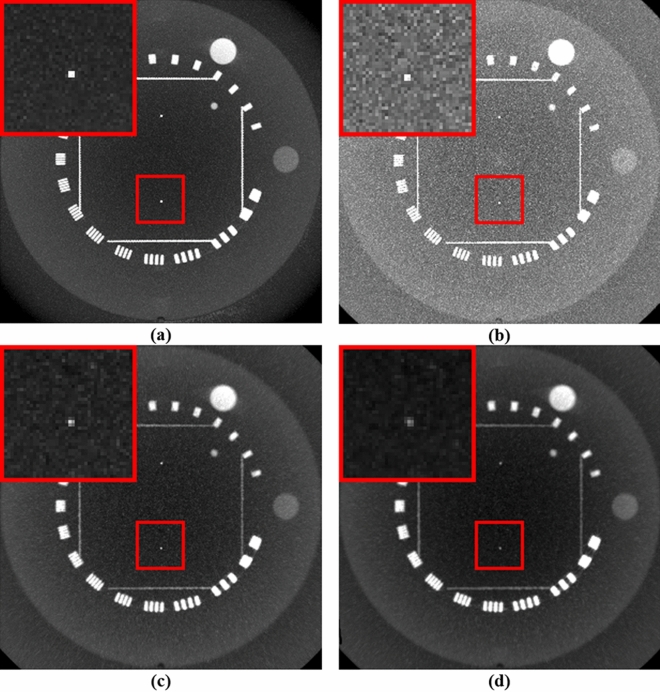
Comparison of MIP of (**a**) high resolution benchmark CBCT images with a high mAs and high resolution CBCT reconstruction images generated by applying FDK algorithms with (**b**) SL + cubic B-spline, (**c**) ATV + cubic B-spline, and (**d**) NLTV + cubic B-spline using Catphan503 phantom acquired under a low mAs. These images are displayed at window (width and level) settings of (1100, 500) HU.

**Figure 9:**
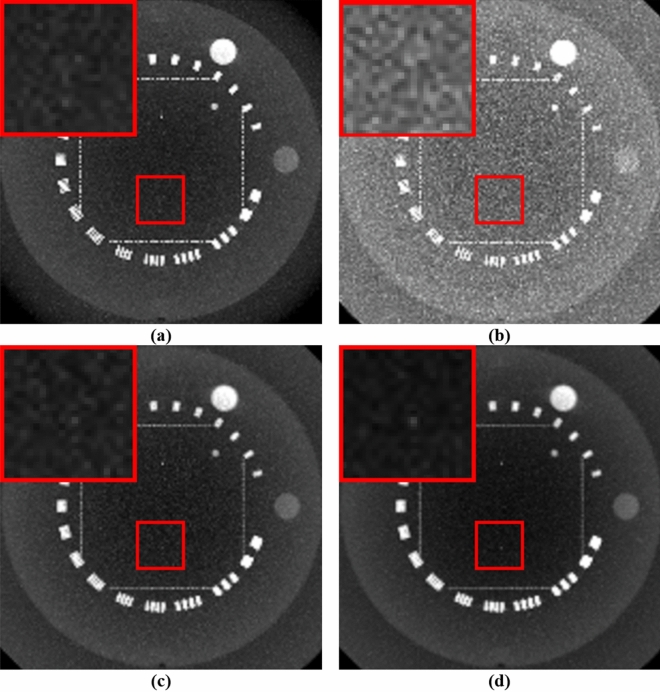
Comparison of MIP of (**a**) low resolution benchmark CBCT images with a high mAs and low resolution CBCT reconstruction images generated by applying FDK algorithms with (**b**) SL + cubic B-spline, (**c**) ATV + cubic B-spline, and (**d**) NLTV + cubic B-spline using Catphan503 phantom acquired under a low mAs. These images are displayed at window (width and level) settings of (1100, 500) HU.

Figures 8 and 9 compare the maximum intensity projections (MIP) of the reconstructed images. The MIP image facilitates extraction of the highest value encountered along the corresponding viewing ray at each pixel of the reconstructed images so that one can determine whether some regions with a high contrast are preserved while reducing the noise. The proposed method showed that regions with remarkable features are well preserved while reducing artifacts in the central region corresponding to the benchmark image compared to the other two algorithms. After adding NLTV, the changes in the homogeneous region were smoother, whereas those in the inhomogeneous regions were almost preserved. For the low resolution CBCT, a thin point feature showing high contrast in the red ROI disappeared, even in the case of the benchmark image. However, it was found that this thin feature was preserved in the reconstructed image when the proposed NLTV denoising was performed.

**Figure 10:**
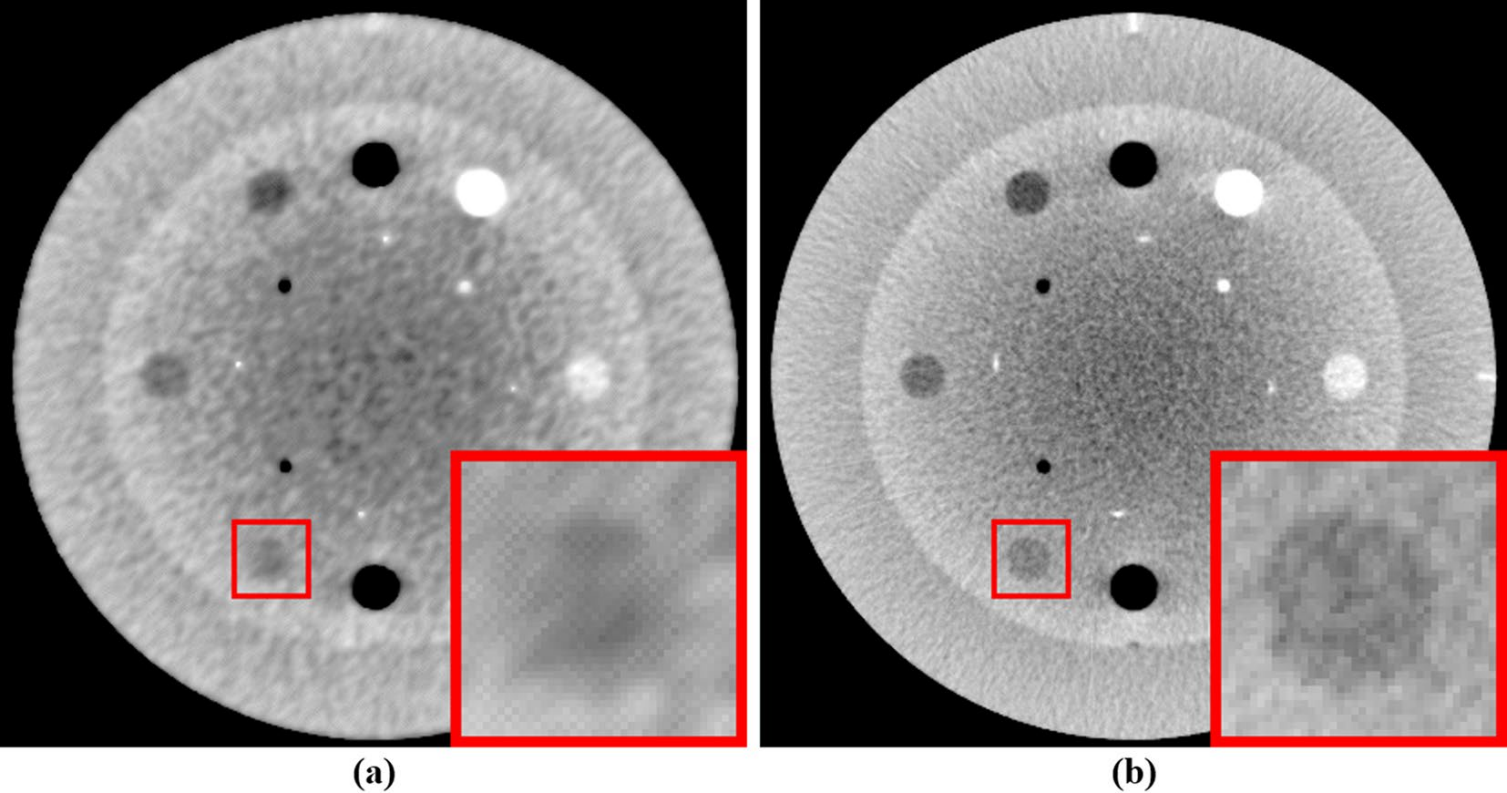
Comparison between high resolution CBCT images generated by applying (**a**) NLTV on FDK reconstructed images as a post-processing strategy and (**b**) FDK with NLTV-denoised projection data as a pre-processing strategy. These Catphan503 phantom images are displayed at window (width and level) settings of (750, 0) HU.

Figure 10 shows a visual inspection of the CBCTs generated by the post-processing approach of applying NLTV to the FDK reconstructed image. In addition, it depicts the proposed method, i.e., the pre-processing approach of applying FDK to the NLTV-denoised projections. We compared the low contrast resolution properties between the two approaches, as in the magnified area. The pre-processing strategy further revealed a higher low contrast detection and detail-preserving effect compared to the post-processing strategy.

## Discussion

CBCT reconstruction from low mAs has been challenging. X-ray tubes produce photons via electron bombardment of a metallic anode. The number of photons is determined by the current across the tube (mA) and the exposure time (s), which are sometimes multiplied together as mAs. Decreasing the mAs decreases the number of electrons hitting the anode, and consequently, the number of photons generated by the tube decreases linearly. This decreases the number of photons reaching the detector, reducing the signal-to-noise ratio (SNR) of the projection data.

The total variation (TV) transform was considered to mitigate the noise imprinted on the projection data, but it tended to keep the edge information too smooth by uniformly penalizing the local image gradient. To overcome this limitation, ATV was proposed to reduce the blurriness at edge regions by deriving the anisotropic edges expressed as the exponential function of the adaptively weighted local image gradient of the projection data. The limitation of ATV is that the capability of deriving local edge information is restricted by the quality of projection data. The projection data acquired at low mAs settings suffer from severe noise. Thus, it is difficult to develop a local edge detection operator that reliably finds features with smaller details and is noise-insensitive. Conversely, the NLTV can help overcome this shortcoming by utilizing more global searches and non-uniform weights depending on the similarities between non-local patches. Combining the cubic B-spline interpolation-based backprojector allows the NLTV denoising-based FDK algorithm to be further enhanced and obtain more precise low-dose CBCT reconstruction images. The reason for such a performance improvement was that the CBCT reconstructed from conventional FDK with SL, ATV, and NLTV were based on nearest neighbor (NN) interpolation-based backprojector. NN is the simplest and fastest interpolation method, but it can introduce significant distortion. The cubic B-spline interpolation is more complex than the NN method and is thus more computationally expensive. For an unknown pixel in the projection data, the influence range is extended to16 adjacent pixels. Then the pixel value is calculated as 16 pixels based on the distance. More complex improved interpolation methods such as cubic B-spline make the interpolation curve smoother. Thus, there are no discrete defects and the high-frequency components are faded. This characteristic provides better visual effects without false artificial traces even at high voxel sizes and low resolutions.

We applied our proposed approach, the combination of NLTV and cube B-spline interpolation, to phantom data and compared its performance with the results from SL and ATV filtrations in terms of visual inspection, RMSE, CNR, and correlation. Reconstructions performed by the proposed method indicated that a higher image quality could be achieved with projection data acquired under a lower mAs protocol. We also determined that the proposed method shows a CNR with a spatial resolution comparable to those of images reconstructed using projections acquired from a high-dose protocol. Moreover, NLTV denoising yields filtered projection data with a greatly reduced noise using non-local patches. The cubic B-spline interpolation process slightly improved the reconstruction accuracy on SL, ATV, and NLTV, as well as the image clarification. All these results indicate that the proposed algorithm not only achieves a potential radiation dose reduction in the repeated CBCT scans, but also eliminates noise effectively without heavily compromising the striking features.

Technically, compared with the ATV transform, the proposed technique might increase the computational cost due to the larger search area when computing the weight function. In this work, to balance the reconstructed image quality with the computational burden, the search area was limited to 21 × 21 pixels around each pixel. The extra computational burden is related to the time elapsed for the backprojector with cubic B-spline interpolation. In our implementation, OpenMP was used to parallelize the proposed algorithm on an Intel Xeon CPU with 24 physical cores and 48 logical processors. Therefore, the proposed algorithm can be extended and accelerated by using a GPU^28^. The number of iterations for the NLTV minimization was determined to be ten, however, this value may not be optimal. Among the datasets used, we observed better reconstructed images with suppressed noise and preserved edges when the number of iterations was ten.

The same magnitude of imaging dose reduction can be achieved by reducing the number of projections while maintaining the mAs setting. However, this approach assumes fast gantry rotation. The maximum gantry speed of clinically used CBCT scanners is still limited to 1 rpm or 6°/s. Thus, low mAs acquisition may be a better option for imaging dose reduction than sparse view acquisition in practical aspects.

The NLTV denoising method can be extended to various applications. It can be used as an image fidelity term in compressed sensing-based approaches^23^. NLTV-based FDK reconstruction images can be used as an initial guess image for iterative reconstruction^10,29^. In general, it has been observed that the better the initial guess image when using an iterative reconstruction algorithm, the faster the convergence and CBCT images that can be created with a high contrast. In addition, a ray-driven backprojector can be combined with NLTV^28^.

In conclusion, an improved FDK reconstruction algorithm for low-dose CBCT imaging has been developed by adding non-local total variation (NLTV) denoising in the filtering process and a voxel-driven backprojector based on cubic-B spline interpolation. The proposed approach allows the conventional FDK algorithm to be used for low mAs image reconstruction without requiring iterative reconstruction algorithms, which are computationally demanding. Moreover, substantial reductions in radiation exposure associated with the low mAs projection acquisition can facilitate wider practical applications of IGRT using daily online CBCT imaging with corrections for setup errors.

## Data availability

All data generated or analyzed during this study are included in the article.
